# Correction: Srisuphanunt et al. Molecular Mechanisms of Antibiotic Resistance and Novel Treatment Strategies for *Helicobacter pylori* Infections. *Trop. Med. Infect. Dis.* 2023, *8*, 163

**DOI:** 10.3390/tropicalmed8090429

**Published:** 2023-08-29

**Authors:** Mayuna Srisuphanunt, Polrat Wilairatana, Nateelak Kooltheat, Thitinat Duangchan, Gerd Katzenmeier, Joan B. Rose

**Affiliations:** 1 Department of Medical Technology, School of Allied Health Sciences, Walailak University, Nakhon Si Thammarat 80160, Thailand; nateelak.ko@wu.ac.th (N.K.); thitinat.du@wu.ac.th (T.D.); 2 Excellent Center for Dengue and Community Public Health, School of Public Health, Walailak University, Nakhon Si Thammarat 80160, Thailand; 3 Department of Clinical Tropical Medicine, Faculty of Tropical Medicine, Mahidol University, Bangkok 10400, Thailand; 4 Hematology and Transfusion Science Research Center, School of Allied Health Sciences, Walailak University, Nakhon Si Thammarat 80160, Thailand; 5 Akkhraratchakumari Veterinary College, Walailak University, Nakhon Si Thammarat 80160, Thailand; gerd.ka@wu.ac.th; 6 Department of Fisheries and Wildlife, Michigan State University, East Lansing, MI 48823, USA; rosejo@msu.edu

In the original publication [1], the funder New Strategic Research Project (P2P), Walailak University, under grant no. CGS-P2P-2565-069, and the health science research grant from Walailak University, Thailand, under grant no. WU-IRG-64-077 awarded to Mayuna Srisuphanunt, were not required. The correct funding appears below. The authors state that the scientific conclusions are unaffected. This correction was approved by the Academic Editor. The original publication has also been updated.

**Funding:** This research received no external funding.
